# The lesion core extent modulates the impact of early perfusion mismatch imaging on outcome variability after thrombectomy in stroke

**DOI:** 10.3389/fneur.2024.1366240

**Published:** 2024-05-22

**Authors:** Maria Marburg, Linda F. Rudolf, Christine Matthis, Alexander Neumann, Constantin Schareck, Hannes Schacht, Robert Schulz, Björn Machner, Peter Schramm, Georg Royl, Philipp J. Koch

**Affiliations:** ^1^Department of Neurology, University Hospital Schleswig-Holstein, Lübeck, Germany; ^2^Department of Neuroradiology, University Hospital Schleswig-Holstein, Lübeck, Germany; ^3^Department of Social Medicine and Epidemiology, University Hospital Schleswig-Holstein, Lübeck, Germany; ^4^Department of Radiology, University Hospital Schleswig-Holstein, Lübeck, Germany; ^5^Department of Neurology, University Medical Center Hamburg Eppendorf, Hamburg, Germany; ^6^Department of Neurology, Schoen Clinic Neustadt, Neustadt in Holstein, Germany; ^7^Center of Brain, Behavior and Metabolism, University of Lübeck, Lübeck, Germany

**Keywords:** stroke, outcome, thrombectomy, perfusion, imaging

## Abstract

**Introduction:**

Despite profitable group effects on functional outcomes after mechanical thrombectomy (MT) in large vessel occlusion (LVO), many patients with successful reperfusion show a non-favorable long-term outcome, highlighting the necessity to identify potential biomarkers predicting outcome variability. In this regard, the role of perfusion mismatch imaging for outcome variability in the early time window within 6 h after symptom onset is a matter of debate. We attempted to investigate under which conditions early perfusion mismatch imaging accounts for variability in functional outcomes after mechanical thrombectomy.

**Patients and methods:**

In a retrospective single-center study, we examined 190 consecutive patients with LVO who were admitted to the Medical Center Lübeck within 6 h after symptom onset, all of whom underwent MT. Perfusion mismatch was quantified by applying the Alberta Stroke Program Early CT score (ASPECTS) on CT-measured cerebral blood flow (CBF-ASPECTS) and subtracting it from an ASPECTS application on cerebral blood volume (CBV-ASPECTS), i.e., ASPECTS mismatch. Using multivariate ordinal regression models, associations between ASPECTS mismatch and modified Rankin Scale (mRS) after 90 days were assessed. Furthermore, the interaction between ASPECTS mismatch and the core lesion volume was calculated to evaluate conditional associations.

**Results:**

ASPECTS mismatch did not correlate with functional outcomes when corrected for multiple influencing covariables. However, interactions between ASPECTS mismatch and CBV-ASPECTS [OR: 1.12 (1.06–1.18), *p*-value < 0.001], as well as NCCT-ASPECTS [OR: 1.15 (1.06–1.25), *p*-value < 0.001], did show a significant association with functional outcomes. Model comparisons revealed that, profoundly, in patients with large core lesion volumes (CBV-ASPECTS < 6 or NCCT-ASPECTS < 6), perfusion mismatch showed a negative correlation with the mRS.

**Discussion and conclusion:**

Perfusion mismatch imaging within the first 6 h of symptom onset provides valuable insights into the outcome variability of LVO stroke patients receiving thrombectomy but only in patients with large ischemic core lesions.

## Introduction

In recent years, the selection of patients with anterior circulation large vessel occlusion (LVO) for mechanical thrombectomy (MT) has been continuously extended. Profitable outcomes have been shown until 24 h after symptom onset (1, 2), and most recently, randomized controlled trials (RCT) have shown a group benefit of MT for patients even with large core lesion volume (1–4). However, there are a significant number of patients with futile recanalization, i.e., poor outcomes despite successful MT. This highlights the urgent need to identify biomarkers that most likely predict a beneficial outcome and therapeutic gain for individual patients after mechanical thrombectomy. In patients presenting with anterior circulation LVO within 6 h after symptom onset, early diagnostics used in most RCT trials includes non-contrast CT (NCCT) and demonstration of the LVO using angiography to select patients for further endovascular treatment (5–7). CT-perfusion (CTP) imaging allows the quantification of irreversibly damaged and potentially salvageable brain tissue, i.e., the mismatch between definite infarction and tissue with reduced perfusion (8–11). Perfusion imaging is not commonly used in early diagnostics within the first 6 h since its primary usage is for penumbra imaging, which, for various reasons like RCT designs, starts at 6 h when considering patients with LVO and stroke. Therefore, it has not been systematically investigated in its relevance for functional outcome variability but instead used to increase the sensitivity depending on the healthcare system (12). Being widely accessible and still essential for patient selection for MT in the extended or unknown time window by estimating targeted mismatch (13), it may add valuable information for patient outcome variability within the first 6 h after symptom onset. By comparing MT patients with a historical cohort without MT, it has been shown that in patients with large ischemic core lesions within the first 6 h of symptom onset, the prevalence of CTP mismatch might distinguish patients who benefit from MT from those who do not (14). Reperfusion in patients with targeted CTP mismatch was associated with better outcomes in patients within 6 h after symptom onset (15) and patients after 6 h of onset, suggesting the potential role of CTP mismatch for clinical decision-making in the early time window (16). On the contrary, a comprehensive meta-analysis evaluated cohort studies within the first 6 h after symptom onset and did not see any association between functional outcomes and initial mismatch, but rather with lesion core volume (17). The perfusion mismatch is most commonly defined on validated cutoffs on specific perfusion maps (e.g., CBF < 30%, Tmax > 6 s) (11). However, its determination relies heavily on post-processing procedures, including smoothing and signal deconvolution typically integrated into commercial software (e.g., RAPID, VizAI) (18), which restricts its applicability in resource-limited settings. We introduce an alternative region-based approach to describe the extent of perfusion mismatch by applying the Alberta Stroke Program Early CT score (ASPECTS) on perfusion imaging maps. It further implies the potential advantage of weighting the perfusion mismatch or extent of the core lesion depending on the specific anatomical region involved compared to a solely volume-based approach. The core lesion was defined based on the CBV for its high correlation with MRI CBV maps within 6 h after onset (19). Tissue at risk was defined based on reduced CBF.

## Aims and hypothesis

The relevance of mismatch and, consequently, the rationale of perfusion imaging within the early time window remains unclear. Arguably, the perfusion mismatch might only be relevant in a specific subgroup of patients with certain clinical features. This aids the development of biomarkers and prediction models aiming at the likelihood of beneficial outcomes and potential therapeutic gain after MT individually.

This study explores the significance of perfusion imaging for functional outcome variability after MT in patients with LVO of the anterior circulation admitted within 6 h of symptom onset.

## Materials and methods

### Patient selection

Patients with anterior circulation stroke due to LVO admitted to the University Medical Center (UKSH) in Lübeck from August 2014 to April 2020 within 6 h of symptom onset were retrospectively assessed. Eligibility was given if patients (i) showed any perfusion deficits in the CTP, (ii) received CT-angiography confirming LVO of the anterior circulation, (iii) underwent MT due to confirmed LVO of the anterior circulation in CT-angiography, and (iv) sufficient clinical information in the medical charts regarding the National Institutes of Health Stroke Scale (NIHSS) at admission and the modified Rankin Scale (mRS) at follow-up after 90 days was given. Patients with isolated occlusion of peripheral branches of the middle cerebral artery (M2 and distal) were excluded. The study was conducted in accordance with the Declaration of Helsinki and approved by the local ethical committee (Nr. 2023-129). As the data were already anonymized, written informed consent was not sought for the present study. The report follows STROBE guidelines for cohort studies.

### Image acquisition and analysis

NCCT, as well as CTP examinations, were acquired on two different scanners. The first scanner was a Siemens Somatom Definition As (Siemens Healthineers, Erlangen, Germany), while the second was a CT6000 iCT from Philips (Philips Healthcare, Hamburg, Germany). In the case of the Siemens scanner, the NCCT was carried out using 100-kV, z-axis dose modulation with a reference tube load of 513 mAs, 1 second resolution time, a pitch set to 0.8, and an effective detector width of 12 mm. However, the kV value was set to 80, and the tube load was referred to 105 mAs at a revolution time of 0.3 s for CTP scans. The CTP acquisition was performed in 45 s with an intrinsic delay of 4 s after bolus injection and a cycle time of 1.5 s with helical image acquisition at 2 × 38, 4-mm detector width, allowing whole brain coverage.

In the case of the Philips scanner, the NCCT was carried out using 100 kV, a fixed tube load of 380 mAs, 0.4 s revolution time, a pitch set to 0.3, and an effective detector width of 40 mm. However, the kV value was set to 80, and the tube load was referred to 100 mAs at a revolution time of 0.33 s for CTP scans. The CTP acquisition was performed in 40 s with an intrinsic delay of 4 s after bolus injection and a cycle time of 1.5 s with static, i.e., axial image acquisition at 80 mm detector width, allowing whole brain coverage.

For postprocessing, syngo.via (Siemens Healthcare, Forchheim) was used with a 5.0 mm slice thickness to generate maps of the CBV and CBF.

The NCCT, as well as perfusion maps (CBV and CBF), were visually inspected by two experienced neuroradiologists (LR and AN) blinded to the clinical outcome. The ASPECTS was applied to NCCT (NCCT-ASPECTS) and the two perfusion maps (CBV-ASPECTS, CBF-ASPECTS) to quantify the extent of early ischemic changes and perfusion deficits. In case of a discrepancy between the two raters, the median was taken. If the median was between two absolute values, the lower value was taken as the extent of the lesion visualized in follow-up MRI is often more extensive than that in initial NCCT-ASPECTS (20). The perfusion mismatch, i.e., the ASPECTS mismatch, was operationalized by CBV-ASPECTS—CBF-ASPECTS values. An alternative approach was performed to estimate the perfusion mismatch volume as well as the core lesion volume based on the perfusion maps CBF (< 30%) and Tmax (>6 s) using an in-house Python script. For a detailed description, please see the Supplementary material.

### Clinical and demographic data

The primary outcome measure was defined as the modified Rankin Scale (mRS) obtained 90 days after stroke. Data on age, National Institute of Health Stroke Scale (NIHSS) at admission, sex, the affected hemisphere (side), and the Thrombolysis in Cerebral Infarction (TICI) score were extracted from the medical charts and the neuroradiological reports. All researchers extracting the clinical information were blinded to the functional outcomes of individual patients.

### Statistical analysis

The association between ASPECTS mismatch and mRS after 90 days was assessed using multivariate ordinal logistic regression models. Odds ratios (OR) with 95% confidence intervals (CI) were fitted for more deficits in patients with larger mismatches. Models were fully adjusted for age, NCCT-ASPECTS, NIHSS at admission, sex, side of the lesion, and TICI score. Furthermore, two interactions were evaluated to explore ASPECTS mismatch information in the dependency from the core lesion volume as operationalized by CBV- and NCCT-ASPECTS. When testing the interaction with CBV-ASPECTS for association with the outcome, CBV-ASPECTS was included as an additional covariable. Further sensitivity analyses were conducted. Separate multivariate ordinal logistic regression models were used (i) for the subgroup of patients with successful recanalization and (ii) corrected for individual medical history. Multivariate imputation analyses were performed to test for the influence of selective bias due to missing functional data, and statistical models were repeated in 216 patients. Multivariate ordinal logistic regression models were used similarly based on the volumetric description of the core lesion and perfusion mismatch using CBF and Tmax (Supplementary material).

Finally, the results of univariate regression modeling assessing the relationship between ASPECTS mismatch and mRS 90 days after stroke were compared in patients with extended core lesions against those with less core lesion sizes. Subgroups included all patients having a score below 6 in the CBV-ASPECTS or NCCT-ASPECTS and patients with a score of 6 or higher. *P*-values were corrected for multiple comparisons. Models were compared using LR statistics. Statistical analyses were performed using Python (v. 3.9.13; Toolboxes used: Scipy, Statsmodels).

## Results

### Clinical and demographic data

**Figure 1:**
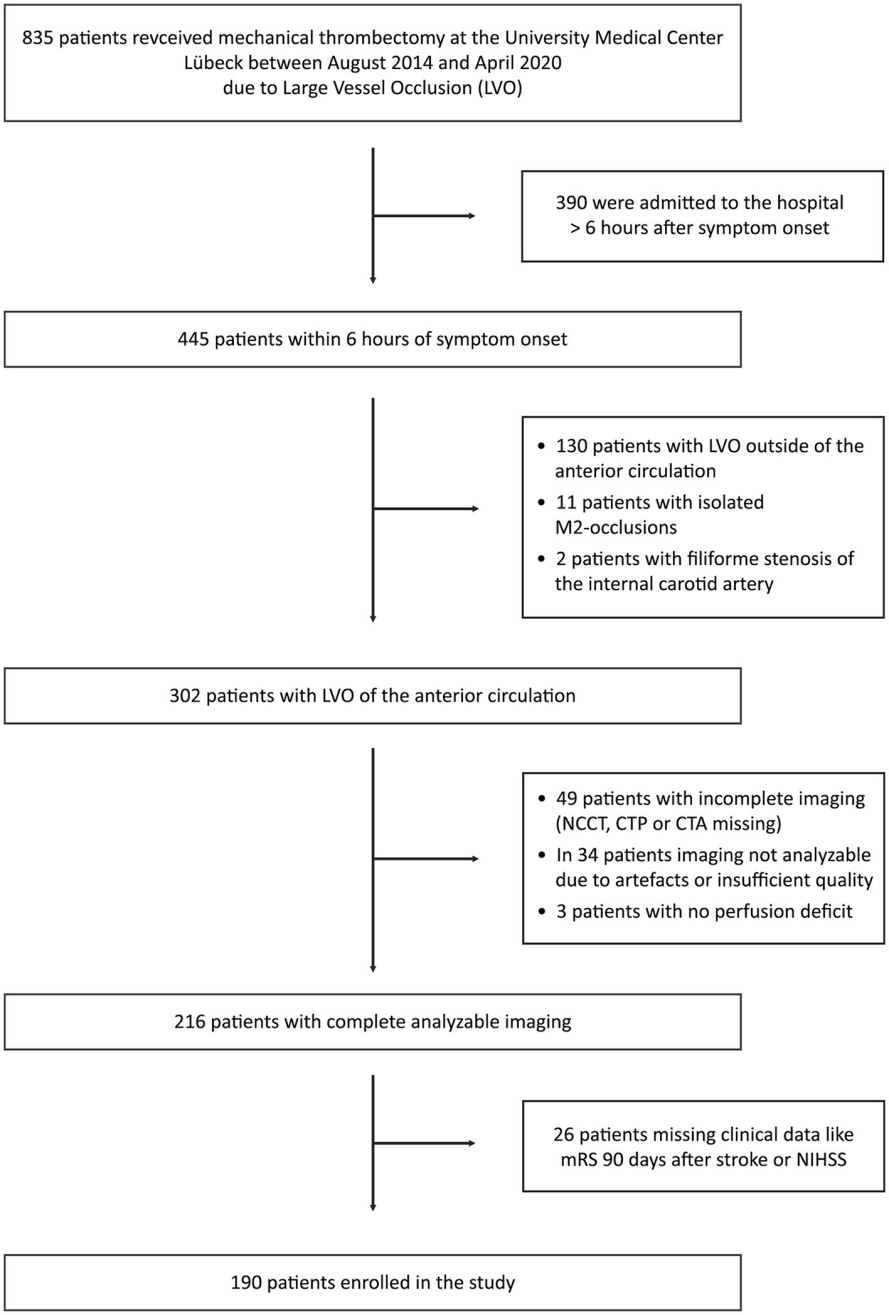
Recruitment. CTA, computed tomography angiography; CTP, computed tomography perfusion; LVO, large cerebral vessel occlusion; mRS, modified Rankin Scale; NCCT, non-contrast computed tomography; and NIHSS, National Institutes of Health Stroke Scale.

Between August 2014 and April 2020, 835 patients were admitted to UKSH Lübeck due to LVO and treated with mechanical thrombectomy. A total of 445 (53%) patients were admitted to the hospital < 6 h after symptom onset, with 302 patients with LVO of the anterior circulation. Complete imaging protocols, including NCCT and perfusion maps with sufficient quality to assess the ASPECTS, were available in 216 patients. Complete clinical and demographical data, including the mRS 90 days after stroke, were accessible in 190 patients included in the final analysis (Figure 1). The same cohort was studied in a separate analysis evaluating white matter disconnectivity based on pre-interventional perfusion imaging (21). The clinical and demographic characteristics of the study cohort are presented in Table 1.

**Table 1:** Patient baseline clinical and demographic characteristics.

**Variable**	***N* = 190**
Age (±SD) in yrs.	72 ± 13
Sex: male in number (proportion %)	93 (50)
Median NIHSS (IQR)	16 (11–18)
Median NCCT-ASPECTS (IQR)	7 (6–9)
Median CBV-ASPECTS (IQR)	6 (4–8)
Median CBF-ASPECTS (IQR)	1 (1–3)
Median CTP mismatch (IQR)	4 (2–6)
Time imaging to reperfusion (min ± SD, *N* = 173)	113 (49)
**Number (proportion %) of patients with**	
Affection of the right hemisphere	97 (51)
IV Thrombolysis received	134 (71)
Successful recanalization (≥TICI 2b)	181 (95)
CBV-ASPECTS < 6	83 (44)
NCCT-ASPECTS < 6	37 (19)
**Vessel occlusion in CTA**	
Proximal ICA	54 (28)
Distal ICA	15 (8)
Distal ICA/Carotis T	23 (12)
M1	105 (55)
M2	1 (1)
**Diagnosis of**	
Arterial hypertension	128 (67)
Diabetes mellitus	40 (21)
Hypercholesterolemia	53 (28)
Atrial fibrillation	85 (45)
History of ischemic stroke	17 (9)

ICA, Internal carotid artery; ASPECTS, Alberta Stroke Program Early CT Score; CBF, cerebral blood flow; CBV, cerebral blood volume; CTP mismatch, CBV-ASPECTS – CBF-ASPECTS; IQR, denotes interquartile range; M1/M2, first and second bifurcation of the arteria cerebri media; NIHSS, National Institutes of Health Stroke Scale ranging from 0-42 indicating the functional deficit; SD standard deviation; TICI 2b, thrombolysis in cerebral infarction indicating successful reperfusion after performed mechanical thrombectomy.

### Association between CTP mismatch and functional outcome

ASPECTS mismatch did not contribute significantly to the outcome variability (Table 2). NCCT-ASPECTS [OR: 0.66 (0.55–0.80), *p*-value < 0.001], age [OR: 1.06 (1.04–1.09), *p*-value < 0.001], sex [OR: 0.45 (0.26–0.79), *p*-value = 0.006], and TICI [OR: 0.53 (0.38–0.74), *p*-value < 0.001] contribute significantly to the functional outcomes with higher NCCT-ASPECTS, lower age, male gender, and higher reperfusion scale being associated with a better functional outcome 90 days after stroke. There was no significant association between perfusion mismatch volume based on CBF and Tmax and functional outcomes (Supplementary Table S1).

**Table 2:** Multivariable ordinal logistic regression: mRS at 90 days after stroke.

**Multivariable ordinal logistic regression model; outcome: mRS 90 days after stroke**		
**Variable**	**OR (95% CI)**	***p*** **-value**
ASPECTS mismatch	0.99 (0.85–1.15)	0.903
ASPECTS mismatch × CBV-ASPECTS	1.12 (1.06–1.18)	< 0.001
ASPECTS mismatch × NCCT-ASPECTS	1.15 (1.06–1.25)	< 0.001

Shown are the results of the three multivariable ordinal logistic regression models evaluating the association between ASPECTS mismatch, the interaction ASPECTS mismatch x CBV-ASPECTS, and the interaction ASPECTS mismatch x NCCT-ASPECTS with mRS 90 days after stroke with given Odds Ratio, 95% confidence interval and *p*-values given for the variable of interest. In all models influencing factors such as NCCT-ASPECTS, age, sex, side, NIHSS, and TICI were included as covariables. In the model assessing the influence of the interaction between ASPECTS mismatch and CBV-ASPECTS, CBV-ASPECTS was further included as a covariable. ASPECTS mismatch, CBV-ASPECTS—CBF ASPECTS; CBV-ASPECTS, Cerebral Blood Volume Alberta Stroke Program Early CT Score; NCCT-ASPECTS, Non-Contrast CT Alberta Stroke Program Early CT Score.

### Evaluating the interaction between ASPECTS mismatch and CBV-ASPECTS as well as between ASPECTS mismatch and NCCT-ASPECTS and its relevance for functional outcomes

The interaction between ASPECTS mismatch and CBV-ASPECTS, as well as the interaction between ASPECTS mismatch with NCCT-ASPECTS, showed a significant contribution to functional outcomes in the multivariate ordinal logistic regression model (Table 2). Conducting the same multivariate ordinal logistic regression modeling among patients achieving successful recanalization (TICI ≥ 2b) yielded consistent findings (Supplementary Table S2). The results were consistent after correcting for the individual medical history (Supplementary Table S3) and after performing multivariate imputation analyses to compensate for missing functional outcome data (*N* = 216, Supplementary Table S4). There was no significant association between the interaction of perfusion mismatch volume and estimates of core lesion extent (CBF < 30%, NCCT-ASPECTS) with functional outcome when considering CBF < 30% and Tmax > 6 s to define perfusion mismatch (Supplementary Table S1).

### Association between ASPECTS mismatch and functional outcome within different subgroups of lesion core volumes

Within the subgroups of patients with CBV-ASPECTS with a score < 6, there was a significant association between ASPECTS mismatch and mRS 90 days after stroke [OR: 0.68 (0.50–0.91), *p*-value = 0.036]. This association was not significant in the group of patients with CBV-ASPECTS of 6 or higher [OR: 1.11 (0.91–1.35), *p*-value = 0.302]. Model comparison using LR statistics favors the model including patients with low CBV-ASPECTS (*p* < 0.001). In patients with NCCT-ASPECTS lower than 6, there is a significant association between ASPECTS mismatch and mRS 90 days after stroke [OR: 0.52 (0.30–0.91), *p*-value = 0.042], which is not present within the group of patients with NCCT-ASPECTS of 6 or higher [OR: 0.97 (0.79–1.03), *p*-value = 0.192]. Model comparison favors the model, including patients with low NCCT-ASPECTS (*p* < 0.001).

**Figure 2:**
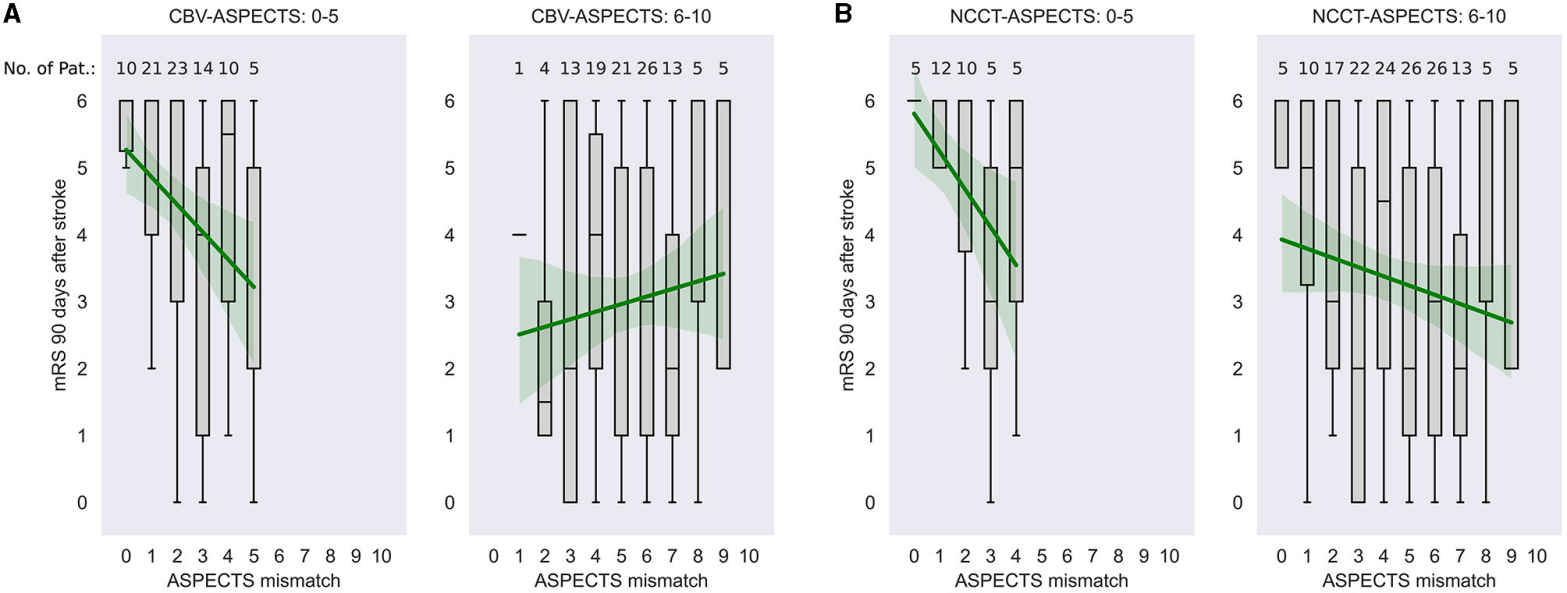
Relationship between ASPECTS mismatch and mRS 90 days after stroke in dependency of CBV-ASPECTS and NCCT-ASPECTS. The mRS scores at 90 days after stroke are illustrated in relation to the ASPECTS mismatch at onset within patients below and equal or above 6 score points in CBV-ASPECTS **(A)** and NCCT-ASPECTS **(B)**. Data are shown with median, first and third quartile, minimum, and maximum given. Numbers above each box plot indicate the number of patients within this subgroup. Furthermore, for illustrative reasons only, data are fit to a linear regression model with a given trend line and 95% confidence Interval (green). CBV-ASPECTS, Cerebral Blood Volume Alberta Stroke Program Early CT Score; ASPECTS mismatch, CBV-ASPECTS—CBF-ASPECTS; NCCT-ASPECTS, Non-Contrast CT Alberta Stroke Program Early CT Score.

Figure 2 illustrates the relationship between ASPECTS mismatch and functional outcomes between the subgroups of patients with CBV-ASPECTS **(A)** and NCCT-ASPECTS **(B)** below and above/equal to 6. Both comparisons show negative correlations between ASPECTS mismatch and mRS 90 days after stroke in the group with lower CBV-ASPECTS or NCCT-ASPECTS.

## Discussion

Within the presented analysis, we aimed to investigate the conditional functional meaning of perfusion imaging in patients with LVO of the anterior circulation receiving mechanical thrombectomy who were admitted to the hospital < 6 h after the symptom onset.

By calculating the ASPECTS based on the CBV and CBF maps, the ASPECTS mismatch was quantified by subtracting CBF-ASPECTS from CBV-ASPECTS. First, multivariate regression models did not show any association between ASPECTS mismatch and functional outcomes, as NCCT-ASPECTS, age, sex, and TICI already explained most variance. However, by incorporating the interaction of ASPECTS mismatch with measurements of the extended core lesion volume, estimated by CBV-ASPECTS or NCCT-ASPECTS, we could show that perfusion mismatch does become relevant for estimating functional outcomes in those patients with extended early ischemic changes.

These results have significant implications for the future development of predictive modeling regarding functional outcomes following MT, emphasizing a shift toward individualized approaches. They further suggest that among acute stroke patients exhibiting extensive signs of irreversible tissue damage, a substantial perfusion mismatch indicates an elevated likelihood of favorable outcomes post-MT. Conversely, the absence of perfusion mismatch in those patients demands careful consideration regarding the decision to perform MT, as the prospects for favorable outcomes are constrained and might not outweigh interventional risks. Therefore, these findings endorse the selective application of perfusion imaging during the early time window to support clinical management.

Following the current guidelines of acute stroke management, in ischemic stroke patients who are admitted to the hospital within 6 h of symptom onset, further perfusion imaging is not required (7, 22) and might even unnecessarily prolong the acute imaging protocol. Still, depending on the healthcare systems, it is often considered to increase sensitivity for distal vessel occlusions that are difficult to detect by CT angiography (12, 23). Most RCTs showing the profitable effect of MT within the early time window did not consider perfusion imaging (5, 6, 24). However, approximately half of the LVO patients treated in the early time window fail to recover despite successful reperfusion (25, 26). Identifying clinical biomarkers that help distinguish patients with favorable outcomes from those without is needed to understand outcome variability following MT and eventually improve clinical management. In this regard, the role of CTP imaging in the early time window is still unclear. Some studies presented an association between perfusion mismatch and functional outcomes in patients admitted within 6 h of symptom onset (15, 27, 28). In contrast, a comprehensive meta-analysis failed to show any association (17). Furthermore, pooled data of the HERMES collaboration comparing RCTs with and without pre-interventional CTP imaging revealed a similar outcome (29) at the group level. In the late time window, a targeted mismatch is still recommended for patient selection for MT (7–9), following penumbral imaging. However, a large multicenter trial revealed no difference in functional outcomes if patients were selected based on NCCT compared to CTP (30), and a recent RCT proved profitable outcome in patients with large infarcts purely selected based on NCCT (31). These results show that perfusion imaging has not become essential for most patients. However, whether it contains valuable information about the functional outcome variability in a specific patient group or on an individual basis and thus still has a high value in acute stroke imaging needs further investigation. Here, developing promising predictive modeling informing the physicians, the patient, and relatives about potential therapeutic gain and functional outcome greatly influences further clinical management. This could develop acute stroke imaging toward a personalized and individualized approach.

In our analysis, the independent association between perfusion mismatch and functional outcome did not show any association with functional outcome, which aligns with previous studies (17, 29). However, in the subgroup of patients with extended lesion core volume, the presented analyses showed a strong association between perfusion mismatch and outcome (Figure 2), as revealed by the significant association of the interaction between ASPECTS mismatch and CBV-ASPECTS or NCCT-ASPECTS with functional outcomes (Table 2). Thus, applying perfusion imaging within 6 h of symptom onset might be helpful in those patients who show signs of large ischemic lesion cores on NCCT (NCCT ASPECTS < 6), as these patients might otherwise be considered not to undergo MT.

Extended core lesion sizes in LVO patients are associated with worse outcomes. Surprisingly, meta-analyses and recent RCT trials have nevertheless shown a profitable group effect in patients with NCCT-ASPECTS ranging from 3 to 5 (1–4, 31). However, of all patients who received endovascular treatment, 51–69% showed an mRS of 4 or higher, indicating an inability to walk, being bedridden, or dead. Further, risk of symptomatic intracranial hemorrhage increases with growing lesion size (14, 32). It is, therefore, a justified effort to identify biomarkers that indicate a high therapeutic gain on an individual level. In patients with extended signs of early ischemic changes, we could show that perfusion mismatch estimation has a high correlation with functional outcomes. In patients with a CBV- or NCCT-ASPECTS below 6, a higher ASPECT mismatch indicated a high likelihood of a better functional outcome, partially achieving favorable outcomes. With every additional perfusion mismatch within areas of the media territory considered in the ASPECTS, patients had half of the risk worsening one point on the mRS 90 days after stroke. On the contrary, patients in the low ASPECTS group, who showed no mismatch, had a very high risk of severe disability or death (Figure 2). This association was not seen in patients with lower core lesion sizes. Therefore, a higher ASPECTS mismatch might foresee a better outcome only in patients with extended core lesions receiving endovascular treatment and might improve predictive modeling of functional outcomes after MT in the future.

Perfusion imaging maps such as CBV and CBF are generated by modeling tracer dynamics and underlying hemodynamic properties. Established estimations of core lesion volume or tissue at risk include the usage of validated cut-offs on such maps (e.g., CBF < 30%) (11). Still, the optimal threshold depends on preprocessing steps like smoothing or signal deconvolution, usually implemented within commercial software that is not broadly accessible and still shows a high risk of overestimation (33). We introduce an alternative region-based approach to describe the extent of core lesion volumes by applying ASPECTS on perfusion imaging maps, independent of any analytical software and selection of specific cutoff values. As for its application on NCCT, it can be performed by any trained physician and is, therefore, easily and quickly accessible in an emergency setting.

Interestingly, analyses aiming to quantify the volume of perfusion mismatch and core lesion, based on CBF < 30% and Tmax >6 s, did not reveal a significant interaction between perfusion mismatch and core lesion extent with functional outcomes as presented. In addition to the previously discussed uncertainty regarding the optimal threshold for defining irreversible tissue damage and tissue at risk, applying the ASPECTS on perfusion maps represents a regional-based weighted approach to define perfusion alterations. Thus, it is plausible that it is not solely the volume itself but the implicated anatomical regions, which most accurately reflect associations with functional outcomes. In this context, we have recently introduced the functional significance of the preserved corticospinal tract based on CTP for functional outcomes after MT (21).

Several limitations must be considered when interpreting the presented analyses.

Ongoing technical developments, including the introduction of novel devices, hold the potential to significantly decrease the time of imaging to reperfusion, consequently enhancing functional outcomes. However, the retrospective analyses presented included patients from 2014 to 2020, thereby constraining the generalizability of these findings. Additional neuroradiological and clinical markers are known to be related to functional outcome which were not included as cofactors in the presented analysis. Here, the collateral status (34), lesion water uptake (35), the time between imaging and reperfusion (36), and affection of essential white matter pathways (21) relate to the functional outcome and might add complexity to the presented statistical models. Furthermore, the premorbid mRS might significantly affect the post-MT outcome and was not accessible in the retrospective clinical data. A total of 26 patients were excluded from the retrospective analyses due to incomplete functional data, including the mRS at 90 days post-stroke, which could introduce a selection bias. Imputation analyses were performed to address missing values, resulting in consistent outcomes and mitigating the potential impact of selection bias on the analyses. In most trials concerning endovascular treatment effects, a less favorable outcome is defined by the mRS. Still, specific neurological deficits dramatically impact the quality of life, which is not reflected by this score. This includes, e.g., aphasia, spatial and sustained attentional deficits, fine motor function, visual deficits, affection of concentration, mood, and sleep. There is growing evidence of overestimating core lesion volume by CT perfusion (37). Thus, core lesion volume approximations by CTP have to be reflected with caution. Furthermore, the inter-rater variability of ASPECTS is high and limits the generalizability of the presented approach (38).

In conclusion, we could show that in the early time window (<6 h after symptom onset) of LVO stroke, the extent of perfusion mismatch relates to the functional outcome only in those patients with signs of extended ischemic core volumes. These results suggest using perfusion imaging in the early time window of < 6 h of symptom onset to support clinical decision-making on MT in patients with low ASPECTS on the initial NCCT. They further deepen the understanding of outcome variability in patients with LVO in the early time window and in patients with signs of extended ischemic core volumes.

## Data availability statement

The raw data supporting the conclusions of this article will be made available by the authors, without undue reservation.

## Ethics statement

The studies involving human participants were reviewed and approved by the Ethics Committee of the University of Lübeck. Written informed consent from the patients/participants or patients/participants' legal guardian/next of kin was not required to participate in this study in accordance with the national legislation and the institutional requirements.

## Author contributions

MM: Data curation, Writing—original draft, Writing—review & editing. LR: Writing—original draft, Writing—review & editing, Formal analysis. CM: Writing—original draft, Writing—review & editing. AN: Writing—original draft, Writing—review & editing, Conceptualization, Formal analysis. CS: Writing—original draft, Writing—review & editing. HS: Methodology, Formal analysis, Writing—review & editing. RS: Writing—original draft, Writing—review & editing. BM: Writing—original draft, Writing—review & editing. PS: Conceptualization, Data curation, Writing—original draft, Writing—review & editing. GR: Conceptualization, Data curation, Writing—original draft, Writing—review & editing. PK: Conceptualization, Data curation, Formal analysis, Funding acquisition, Investigation, Methodology, Project administration, Resources, Software, Supervision, Validation, Visualization, Writing—original draft, Writing—review & editing.
