# Supplementary material for: The lesion core extent modulates the impact of early perfusion mismatch imaging on outcome variability after thrombectomy in stroke
Source: Front Neurol. 2024 May 22;15:1366240. doi: 10.3389/fneur.2024.1366240 (PMC11150589; doi:10.3389/fneur.2024.1366240)
Supplement: Supplementary file 1 [file Data_Sheet_1.pdf]

Supplementary Online Material:

The lesion core extent modulates the impact of early perfusion mismatch imaging on outcome variability after thrombectomy in stroke

Maria Marburg[1,a], MD, Linda F. Rudolf[2,a], MD, Christine Matthis[3], MD, Alexander Neumann[2], MD, Constantin Schareck[4], MSc, Hannes Schacht[2], MD, Robert Schulz[5], MD, Björn Machner[6], MD, Peter Schramm[2], MD, Georg Royl[1,7], MD, Philipp J. Koch[1,7*], MD

1 Department of Neurology, University Hospital Schleswig-Holstein, Campus Lübeck, Ratzeburger Allee 160, 23538, Lübeck, Germany
2 Department of Neuroradiology, University Hospital Schleswig-Holstein, Campus Lübeck, Ratzeburger Allee 160, 23538, Lübeck, Germany
3 Department of Social Medicine and Epidemiology, University Hospital Schleswig-Holstein, Campus Lübeck, Ratzeburger Allee 160, 23538, Lübeck, Germany
4 Department of Radiology, University Hospital Schleswig-Holstein, Campus Lübeck, Ratzeburger Allee 160, 23538, Lübeck, Germany
5 Department of Neurology, University Medical Center Hamburg Eppendorf, Martinistr. 52, 20246 Hamburg, Germany
6 Department of Neurology, Schoen Clinic Neustadt, Am Kiebitzberg 10, 23730, Neustadt in Holstein, Germany
7 Center of Brain, Behavior and Metabolism (CBBM), University of Lübeck, Ratzeburger Allee 160, 23562, Lübeck, Germany

a These authors contributed equally.
*Corresponding author

Dr. med. Philipp J. Koch
phil.koch@uni-luebeck.de
Department of Neurology
University Hospital Schleswig-Holstein, Campus Lübeck,
23538 Lübeck, Germany

Defining Perfusion mismatch volume and core lesion volume based on CBF and Tmax

Masks for tissue at risk based on Tmax and masks for core lesions based on CBF were created using an in-house Python script. Perfusion maps of CBF and Tmax were created using syngo.via (Siemens Healthcare, Forchheim) with 5.0mm slice thickness. Next, intensity values of the CTP base images were transformed, elevating the tissue contrast, as suggested previously(1). The preprocessed CT images were then coregistered to Montreal Neurological Institute (MNI) standard space with an affine transformation with mutual information as an optimization metric using Advanced Normalization Tools (ANTs; https://github.com/ANTsX). Those transformation matrices were applied to transform the CBF and Tmax maps to the MNI space accordingly. This step was necessary to ensure alignment of the Field of View Center and automatically define healthy and affected hemispheres. Within the affected hemisphere, the core lesion was defined as values <30% of the unaffected hemisphere(2). Tissue at risk was defined as values > 6 seconds within the affected hemisphere. To further reduce noise, small clusters of voxels (<0.2ml) were identified and unmasked using Density-Based Spatial Clustering of Applications with Noise (DBSCAN) as introduced recently(3). The perfusion mismatch volume was defined as those voxels masked as tissue at risk but not as core lesions.

Multivariable regression models were performed to assess the influence of perfusion mismatch volume and functional outcome as well as the interaction of perfusion mismatch volume and core lesion volume as well as NCCT-ASPECTS with functional outcome (Table 1). All models were corrected for the influence of NCCT-ASPECTS, Age, sex, affected side, NIHSS at onset, and TICI.

**Multivariable Ordinal Logistic Regression Model; Outcome: mRS 90 days after stroke**		
Perfusion mismatch volume and Core lesion volume (CBF<30%, Tmax>6sec.)		
Variable	OR (95% CI)	p-value
Perfusion mismatch volume	0.70 (0.43-1.15)	0.158
Perfusion mismatch volume x Core lesion volume	0.71 (0.27-1.86)	0.488
Perfusion mismatch volume x NCCT-ASPECTS	0.88 (0.64-1.21)	0.432

Table S1 | Multivariable Ordinal Logistic Regression: mRS at 90 days after stroke

Shown are the results of the three multivariable ordinal logistic regression models evaluating the association between Perfusion mismatch volume, the interaction Perfusion mismatch volume x Core lesion volume, and the interaction Perfusion mismatch volume x NCCT-ASPECTS with mRS 90 days after stroke with given Odds Ratio, 95% Confidence Interval and p-values given for the variable of interest. In all models influencing factors such as NCCT-ASPECTS, Age, Sex, Side, NIHSS, and TICI were included as covariables. In the model assessing the influence of the interaction between Perfusion mismatch volume and Core lesion volume, Core lesion volume was further included as a covariable. Perfusion mismatch volume and Core lesion volume are based on CBF<30% and Tmax > 6sec. NCCT-ASPECTS: Non-Contrast CT Alberta Stroke Program Early CT Score.

**Multivariable Ordinal Logistic Regression Model; Outcome: mRS 90 days after stroke**		
All patients with successful recanalization (≥TICI 2b, N=181)		
Variable	OR (95% CI)	p-value
ASPECTS mismatch	0.97 (0.83-1.14)	0.730
ASPECTS mismatch x CBV-ASPECTS	1.12 (1.06-1.18)	<0.001
ASPECTS mismatch x NCCT-ASPECTS	1.14 (1.05-1.23)	0.002

Table S2 | Multivariable Ordinal Logistic Regression: mRS at 90 days after stroke

Shown are the results of the three multivariable ordinal logistic regression models evaluating the association between ASPECTS mismatch, the interaction ASPECTS mismatch x CBV-ASPECTS, and the interaction ASPECTS mismatch x NCCT-ASPECTS with mRS 90 days after stroke with given Odds Ratio, 95% Confidence Interval and p-values given for the variable of interest. Only those patients were considered with successful recanalization defined as TICI≥2b. In all models influencing factors such as NCCT-ASPECTS, Age, Sex, Side, NIHSS, and TICI were included as covariables. In the model assessing the influence of the interaction between ASPECTS mismatch and CBV-ASPECTS, CBV-ASPECTS was further included as a covariable. ASPECTS mismatch: CBV-ASPECTS – CBF ASPECTS; CBV-ASPECTS: Cerebral Blood Volume Alberta Stroke Program Early CT Score ; NCCT-ASPECTS: Non-Contrast CT Alberta Stroke Program Early CT Score.

Multivariable Ordinal Logistic Regression Model; Outcome: mRS 90 days after stroke		
Corrected for medical history		
Variable	OR (95% CI)	p-value
ASPECTS mismatch	1.00 (0.86-1.17)	0.962
ASPECTS mismatch x CBV-ASPECTS	1.13 (1.07-1.19)	<0.001
ASPECTS mismatch x NCCT-ASPECTS	1.15 (1.01-1.24)	0.001

Table S3 | Multivariable Ordinal Logistic Regression: mRS at 90 days after stroke

Shown are the results of the three multivariable ordinal logistic regression models evaluating the association between ASPECTS mismatch, the interaction ASPECTS mismatch x CBV-ASPECTS, and the interaction ASPECTS mismatch x NCCT-ASPECTS with mRS 90 days after stroke with given Odds Ratio, 95% Confidence Interval and p-values given for the variable of interest. In all models, influencing factors such as NCCT-ASPECTS, Age, Sex, Side, NIHSS, TICI, as well as aspects of the medical history including diabetes mellitus, arterial hypertension, atrial fibrillation, hypercholesterolemia and history of ischemic stroke were included as covariables. In the model assessing the influence of the interaction between ASPECTS mismatch and CBV-ASPECTS, CBV-ASPECTS was further included as a covariable. ASPECTS mismatch: CBV-ASPECTS – CBF ASPECTS; CBV-ASPECTS: Cerebral Blood Volume Alberta Stroke Program Early CT Score ; NCCT-ASPECTS: Non-Contrast CT Alberta Stroke Program Early CT Score.

Imputation analyses to compensate for missing functional data.

Within the patient selection for the presented retrospective analyses, 26 patients had to be excluded due to missing functional data, such as the NIHSS at onset or the mRS 90 days after stroke. To correct for potential selection bias, multivariate feature imputation was performed to estimate missing values based on age, sex, NIHSS, and mRS at onset and the affected hemisphere. The IterativeImputer class was used and implemented in Python scikit. Multivariable Ordinal Logistic Regression Models were repeated with a total of 216 patients (Table S4).

Multivariable Ordinal Logistic Regression Model; Outcome: mRS 90 days after stroke		
Imputation of missing functional data (mRS, NIHSS, N=216)		
Variable	OR (95% CI)	p-value
ASPECTS mismatch	1.02 (0.90-1.15)	0.771
ASPECTS mismatch x CBV-ASPECTS	1.07 (1.03-1.12)	<0.001
ASPECTS mismatch x NCCT-ASPECTS	1.07 (1.01-1.13)	0.019

Table S4 | Multivariable Ordinal Logistic Regression: mRS at 90 days after stroke

Shown are the results of the three multivariable ordinal logistic regression models evaluating the association between ASPECTS mismatch, the interaction ASPECTS mismatch x CBV-ASPECTS, and the interaction ASPECTS mismatch x NCCT-ASPECTS with mRS 90 days after stroke with given Odds Ratio, 95% Confidence Interval and p-values given for the variable of interest. Multivariable feature imputation was performed to compensate for missing values. In all models, influencing factors such as NCCT-ASPECTS, Age, Sex, Side, NIHSS, and TICI were included as covariables. In the model assessing the influence of the interaction between ASPECTS mismatch and CBV-ASPECTS, CBV-ASPECTS was further included as a covariable. ASPECTS mismatch: CBV-ASPECTS – CBF ASPECTS; CBV-ASPECTS: Cerebral Blood Volume Alberta Stroke Program Early CT Score ; NCCT-ASPECTS: Non-Contrast CT Alberta Stroke Program Early CT Score.

References:

1. Rorden C, Bonilha L, Fridriksson J, Bender B, Karnath H-O. Age-specific CT and MRI templates for spatial normalization. Neuroimage. 2012 Jul;61(4):957–65.
2. Campbell BC V, Christensen S, Levi CR, Desmond PM, Donnan GA, Davis SM, et al. Cerebral blood flow is the optimal CT perfusion parameter for assessing infarct core. Stroke. 2011 Dec;42(12):3435–40.
3. Koch PJ, Rudolf LF, Schramm P, Frontzkowski L, Marburg M, Matthis C, et al. Preserved Corticospinal Tract Revealed by Acute Perfusion Imaging Relates to Better Outcome After Thrombectomy in Stroke. Stroke. 2023;54(12):3081–9.
